# Influence of 37 Years of Nitrogen and Phosphorus Fertilization on Composition of Rhizosphere Arbuscular Mycorrhizal Fungi Communities in Black Soil of Northeast China

**DOI:** 10.3389/fmicb.2020.539669

**Published:** 2020-09-08

**Authors:** Qingfeng Wang, Mingchao Ma, Xin Jiang, Dawei Guan, Dan Wei, Fengming Cao, Yaowei Kang, Changbin Chu, Shuhang Wu, Jun Li

**Affiliations:** ^1^Eco-environmental Protection Research Institute, Shanghai Academy of Agricultural Sciences, Shanghai, China; ^2^Institute of Agricultural Resources and Regional Planning, Chinese Academy of Agricultural Sciences, Beijing, China; ^3^The Institute of Soil Fertility and Environmental Sources, Heilongjiang Academy of Agricultural Sciences, Harbin, China; ^4^College of Life Science, Zhaoqing University, Zhaoqing, China

**Keywords:** rhizosphere, arbuscular mycorrhizal fungi, nitrogen and phosphorus fertilization, community composition, long-term field experiment

## Abstract

Increased inorganic nitrogen (N) and phosphorus (P) additions expected in the future will endanger the biodiversity and stability of agricultural ecosystems. In this context, a long-term fertilizer experiment (37 years) was set up in the black soil of northeast China. We examined interaction impacts of elevated fertilizer and host selection processes on arbuscular mycorrhizal fungi (AMF) communities in wheat rhizosphere soil using the Illumina MiSeq platform. The soil samples were subjected to five fertilization regimes: no fertilizer (CK) and low N (N_1_), low N plus low P (N_1_P_1_), high N (N_2_), and high N plus high P (N_2_P_2_) fertilizer. Long-term fertilization resulted in a significant shift in rhizosphere soil nutrient concentrations. The N fertilization (N_1_ and N_2_) did not significantly change rhizosphere AMF species diversity, but N plus P fertilization (N_1_P_1_ and N_2_P_2_) decreased it compared with CK. Non-metric multidimensional scaling showed that the rhizosphere AMF communities in CK, N_1_, N_2_, N_1_P_1_ and N_2_P_2_ treatments were distinct from each other. The AMF communities were predominantly composed of Glomeraceae, accounting for 30.0–39.1% of the sequences, and the relative abundance of family Glomeraceae was more abundance in fertilized soils, while family Paraglomeraceae were increased in N_1_ and N_2_ compared with CK. Analysis shown that AMF diversity was directly affected by soil C:P ratio but indirectly affected by plant under long-term fertilization. Overall, the results indicated that long-term N and P fertilization regimes changed rhizosphere AMF diversity and community composition, and rhizosphere AMF diversity was both affected by soil C:P ratio and plant.

## Introduction

The interactions between microorganisms belowground and plants aboveground significantly influence ecosystem properties and processes. Arbuscular mycorrhizal fungi (AMF) are mutualistic fungi that form symbiotic relationships with the majority of land plants, including many crops. Within this symbiosis, AMF can provide plants with critical nutrients and water, as the large extra-radical mycelium exploits the soil and transports these nutrients to plants (Hodge and Fitter, 2010). In other cases, AMF can act as protectants against phytopathogens and enhance the sustainability of ecosystems by improving soil structure (Camenzind et al., 2014). The plant, in turn, provides AMF with an energy source, especially carbon (C).

Nutrient depositions by anthropogenic activities have increased in numerous agricultural lands. This elevated nutrient availability would no doubt influence AMF communities, as it could reduce the benefit provided by these symbionts. According to the functional equilibrium model, when soil nutrients become less limited, plants then allocate resources toward compositions of what could acquire other limited resources (Ericsson, 1995) resulting in a decrease in mycorrhizal structures and fine roots (Johnson, 2010). In addition, fungus itself can be nutrient-limited and sensitive to changes in soil properties (Zhou et al., 2016). Studies have showed that elevated concentrations of soil nitrogen (N) as a result of N fertilization decreased AMF biomass, species richness and diversity in some ecosystems (Egerton-Warburton et al., 2007; Verbruggen et al., 2012; Williams et al., 2017). Furthermore, changes to AMF communities caused by N application may be linked to soil P availability (Egerton-Warburton et al., 2007; Cheng et al., 2013). The rhizosphere is considered to be one of the most dynamic interfaces on Earth (Wang et al., 2018). Rhizosphere microbial communities are shaped by interactions between agricultural management and host selection processed (Schmidt et al., 2019), but those studies only focus on the soil or root AMF communities, not on rhizosphere soil. Wang et al. (2019) shown that the root-feeding AMF abundance was increased under elevated N-addition, although other studies found that N decreased its abundance (Jiang et al., 2018). Those results indicated that the rhizosphere AMF may responsed differently to nutrients addition. However, our understanding of how fertilizer and host selection processes influence on this important mutualistic fungi is still limited, and understanding the shifts in rhizosphere AMF structure and composition following long-term fertilization may have significant implications for using the AMF to increase nutrient availability in soils.

Chinese black soil is distributed in a narrow and long area of ∼6 million ha, and 70% of this is in Heilongjiang Province of Northeast China. The relatively high organic matter and cation exchange capacity and its favorable macronutrient status, as well as a thick (60 cm) mollic epipedon, provides favorable soil structure and conditions for plant growth (Xing et al., 2005). The black soil region contributes to up to 30% of the national staple food using only 20% of the national arable land. Thus, it is important for China’s food security. However, extensive agricultural intensification has input large amounts of inorganic fertilizers into this region, resulting in serious degradation of soil physicochemical properties and environmental health since the 1950s (Yin et al., 2015). Zhou et al. (2016) showed that long-term inorganic fertilizer use had significantly decreased soil pH and increased nutrient availability. This nutrient-based alteration significantly changed soil microorganism communities (Yin et al., 2015; Zhou et al., 2016). Our previous study showed that long-term N and P fertilization in black soil reduced fungal diversity and increased ITS gene copy numbers, thus altering the fungal community composition – such shifts were correlated with soil pH (Zhou et al., 2016). However, knowledge of the influences of long-term inorganic fertilizer application on AMF communities and the primary factors driving AMF species diversity in black soils of northeast China is still limited.

To test potential impacts of future nutrient depositions on AMF communities and diversity in black soil, a field experiment using different N and P fertilizer rates was begun in 1980 in Harbin city, northeast China (Wei et al., 2008). In the present study, we investigated the response of rhizosphere AMF species diversity and community composition to different fertilization strategies, and determined the relationships between AMF species diversity, dominant groups and soil parameters associated with these changes. It was hypothesized that long-term N and P fertilizer application would significantly change soil AMF community composition, and AMF species diversity would decrease with the incorporation of N and P fertilization, but increase with N fertilization alone.

## Materials and Methods

### Experimental Description

The long-term field experiment was established in 1980 at the Scientific Observation Station of Arable Land Conservation and Agriculture Environment of Heilongjiang Academy of Sciences (45°40′N, 126°35′E and altitude 151 m), where the mean annual temperature and average annual precipitation of 3.5°C and 533 mm, respectively. The experimental field contains black soil, which is widespread in the Northeast China. The long-term fertilization experiment has a completely randomized block design with three replicated plots (9 m × 4 m). As previous studies shown that AMF composition and diversity was most affected by N and P fertilizers, but not K fertilizer (Lin et al., 2012; Dueñas et al., 2020), we selected the following treatments: no added fertilizer (CK) and low N (N_1_, 150 kg urea ha^–1^ y^–1^), low N plus low P (N_1_P_1_, 150 kg urea plus 75 kg P_2_O_5_ ha^–1^ y^–1^), high N (N_2_, 300 kg urea ha^–1^ y^–1^) and high N plus high P (N_2_P_2_, 300 kg urea plus 150 kg P_2_O_5_ ha^–1^ y^–1^) fertilizer. The N fertilizer was applied as in the form of urea, while the P fertilizer was ammonium hydrogen phosphate and calcium super phosphate. The rates of low N and low P fertilizers in the experimental field follow local customs. Fertilizer treatments were maintained in the same plot location each year. The cropping system was a wheat–maize–soybean rotation, which is one of the main cropping patterns in this area. More detail on this long-term experimental field is given in Wei et al. (2008). The sampling time was during wheat florescence on 29 June 2016. For each replicated plot, roots were taken from 20 randomly selected plants, loosely adhering soil was shaken off and the tightly adhering soil carefully collected. These rhizosphere soils were pooled to form one composite sample. A total of 15 fresh samples (three replicates × five treatments) were transported to the laboratory on ice, and sieved through a 2-mm mesh to remove plant roots. Each sample was divided into two parts: one stored at room temperature for chemical analysis and the other stored at −80°C for molecular analysis. The wheat grain yields were measured after harvest.

### Chemical Analysis

Soil pH was measured with a glass combination electrode using a soil to water ratio of 1:1. Soil available P (AP) and available potassium (AK) were determined according to Olsen (1954) and Helmke and Sparks (1996), respectively. Nitrate (NO_3_^–^)-N and ammonium (NH_4_^+^)-N were extracted with 2M KCl, and determined with a flow injection autoanalyzer (FLA star 5000 Analyzer, Foss, Denmark). Total organic C (TOC) content was determined by wet digestion using a mixture of potassium dichromate and sulfuric acid under heating. Total N (TN) was measured according to Strickland and Sollins (1987).

### Total DNA Extraction and Sequencing

We extracted total DNA from 0.25 g of soil using MoBio Power Soil DNA isolation kits according to the manufacturer’s protocol (MOBIO Laboratories Inc., Carlsbad, CA, United States). To minimize the DNA extraction bias, we combined six successive DNA lots extracted from the same soil sample and purified using a DNeasy Tissue kit (Qiagen, Valencia, CA, United States).

Amplicons of AMF libraries were produced from each of the 15 soil extracts by nested PCR with first-round PCR primers of LR1 (5′-GCATATCAATAAGCGGAGGA-3′) and FLR2 (5′-GTCGTTTAAAGCCATTACGTC-3′) (Van Tuinen et al., 1998), and second-round PCR primers FLR3 (5′-TTGAAAGGGAAACGATTGAAGT-3′) and (5′-TACGTCAACATCCTTAACGAA-3′) (Gollotte et al., 2004). The second-round primers were tagged with sequencing adapters followed by an 8-mer multiplexing identifier. The two rounds of PCR (50 μl) both contained 5 μl of 10× *Pyrobest* Buffer, 4 μl of dNTPs (2.5 mM), 2 μl of each primer (10 μM), 0.75 U of Pyrobest DNA Polymerase and 30 ng of template DNA. The PCR amplification procedure for both rounds was 5 min at 95°C, followed by 30 cycles of 45 s at 95°C (denaturation), 50 s at 58°C (annealing) and 45 s at 72°C (extension), with a final extension step of 10 min at 72°C. PCR products from each sample were purified, pooled together in equimolar ratios and sequenced using the Illumina MiSeq platform. Raw sequence data for all the samples were uploaded to the NCBI Sequence Read Archive under accession number SRX3008208.

### Bioinformatic Analysis of Sequence Data

Sequence read analysis was carried out using Mothur 1.33 (Schloss et al., 2009). The primers and multiplexing identifier were trimmed, and assignment of samples was based on unique barcodes. Reads with a quality score < 20, with ambiguous nucleotides, lacking a complete barcode or <200 bp were removed and excluded from further analysis. This was followed by checking for chimeras and removal of predicted chimeras. The remaining high-quality sequences were then clustered into operational taxonomic units (OTUs) at 97% identity threshold using UPARSE (Edgar, 2013). The longest sequence from each OTU was selected as the representative sequence, and a manual BLASTing against the GenBank non-redundant nucleotide database was used to detect non-Glomeromycota sequences. The non-Glomeromycota sequences and OTU singletons were removed (Camenzind et al., 2014; Williams et al., 2017). The Chao1 index was used to determine the AMF richness, and the phylogenetic diversity index was used to determine the phylogenetic diversity. The AMF richness and phylogenetic diversity were calculated after subsampling according to the sample with the least sequences in Mothur (Schloss et al., 2009).

### Statistical Analysis

Significant differences in soil properties, alpha-diversity and AMF community abundance among samples were determined with a one-way ANOVA and least significance difference (LSD) using SPSS (version 19.1) statistical software (SPSS, Chicago, IL, United States). Pearson’s correlation coefficients were used to assess relationships among soil properties, alpha-diversity and abundant AMF genera. In all tests, *P* < 0.05 was considered to be statistically significant. Non-metric multidimensional scaling (NMDS) was performed to examine the effects of fertilization on the AMF community using CANOCO 5.0. Redundancy analysis (RDA) was carried out using CANOCO 5.0 to determine correlations between environmental variables (pH, AP, AK, TOC, and soil C:P ratio) and AMF community composition with the Monte Carlo permutation test (999 permutations).

Structural equation modeling (SEM) was applied to gain a mechanistic understanding of how soil properties and plant mediate alterations in rhizosphere AMF diversity and composition under different fertilizer regimes. The community composition of AMF was obtained by NMDS, and the NMDS1 were used in the subsequent SEM analysis. SEM analysis was performed with the specification of conceptual model of hypothetical relationships (Supplementary Figure S1), assuming that long-term fertilization alters soil properties and plant biomass, which in turn affects AMF diversity, composition and plant. The maximum likelihood estimation method was used to test the data were fitted to the models, and adequate model fits were indicated by the low χ^2^/df (<2), non-significant chi-square test (*P* > 0.05), a low RMSEA (RESEA < 0.05), and high goodness-of-fit indes (GFI > 0.9) (Zeng et al., 2016; Ning et al., 2020).

## Results

### Long-Term Fertilization Changed Soil Parameters in Wheat Rhizosphere

After a 37-year application of N and P fertilizer, soil properties in the rhizosphere were significantly altered (*P* < 0.05, Table 1). Soil contents of TN and NO_3_^–^-N was not significantly changed by N_1_ and N_1_P_1_, but significantly increased by N_2_ and N_2_P_2_. However, 37 years of fertilization significantly decreased AK, and soil pH steadily decreased from 6.81 to 5.45 with increasing fertilizer inputs. Soil AP was not significantly changed by N fertilization (N_1_ and N_2_), but significantly increased by N plus P addition. The soil C:P ratio in the N_1_ treatment was 43.2% higher than CK, and correspondingly for the N_1_P_1_, N_2_, and N_2_P_2_ treatments was 32.8, 79.1, and 92.1% lower. Furthermore, wheat yield was significantly increased by long-term fertilization, but there were no significant differences between N_1_ and N_2_ or between N_1_P_1_ and N_2_P_2_ treatments.

**TABLE 1:** Properties of soil under different fertilizer treatments.

Variable	CK	N_1_	N_1_P_1_	N_2_	N_2_P_2_	P
pH (1:1H_2_O)	6.81 ± 0.01^a^	6.29 ± 0.07^b^	6.11 ± 0.05^b^	5.72 ± 0.07^c^	5.45 ± 0.13^c^	<0.001
AK (mg kg^–1^)	127.2 ± 6.9^a^	106.4 ± 14.6^ab^	111.6 ± 0.7^ab^	97.4 ± 13.5^b^	86.3 ± 3.9^b^	0.004
AP (mg kg^–1^)	10.36 ± 2.73^c^	7.59 ± 1.74^c^	51.82 ± 11.48^b^	15.68 ± 2.09^c^	136.26 ± 25.26^a^	<0.001
NO_3_^–^-N (mg kg^–1^)	0.93 ± 0.34^b^	2.08 ± 0.26^b^	0.67 ± 0.10^b^	6.38 ± 0.88^a^	6.23 ± 0.67^a^	<0.001
NH_4_^+^-N (mg kg^–1^)	1.41 ± 0.03^b^	18.90 ± 3.35^bc^	14.75 ± 0.37^c^	25.69 ± 4.61^ab^	31.54 ± 3.52^a^	<0.001
TN (g kg^–1^)	1.41 ± 0.03^b^	1.42 ± 0.02^b^	1.39 ± 0.02^b^	1.53 ± 0.07^a^	1.49 ± 0.01^b^	0.003
TOC (g kg^–1^)	22.74 ± 0.15^b^	24.07 ± 0.34^a^	23.80 ± 0.24^ab^	24.02 ± 0.46^a^	23.94 ± 0.71^a^	0.017
C:N	16.16 ± 0.37^ab^	16.93 ± 0.47^a^	15.56 ± 0.76^b^	17.24 ± 0.37^a^	16.09 ± 0.46^ab^	0.013
C:P	2287 ± 522^ab^	3277 ± 672^a^	1537 ± 210^bc^	477 ± 94^cd^	180 ± 35^d^	<0.001
WY (kg ha^–1^)	1546 ± 96^c^	2170 ± 220^b^	3595 ± 267^a^	2188 ± 209^b^	3135 ± 431^a^	<0.001

*Values are mean ± standard deviation (N = 3). Values within the same column followed by the different letters indicate significant difference (P < 0.05). Fertilizer regimes: CK (without fertilizer), N_1_ (150 kg N ha^–1^), N_1_P_1_ (150 kg N ha^–1^ plus 75 kg P ha^–1^), N_2_ (300 kg N ha^–1^), and N_2_P_2_ (300 kg N ha^–1^ plus 150 kg P ha^–1^). Soil properties indicated include AP (available P), AK (available K), TOC (total organic carbon), TN (total N), C:N (TOC:TN), C:P (TOC:AP), and WY (wheat yield).*

### Long-Term Fertilization Changed Rhizosphere AMF Community Composition and Diversity

**FIGURE 1:**
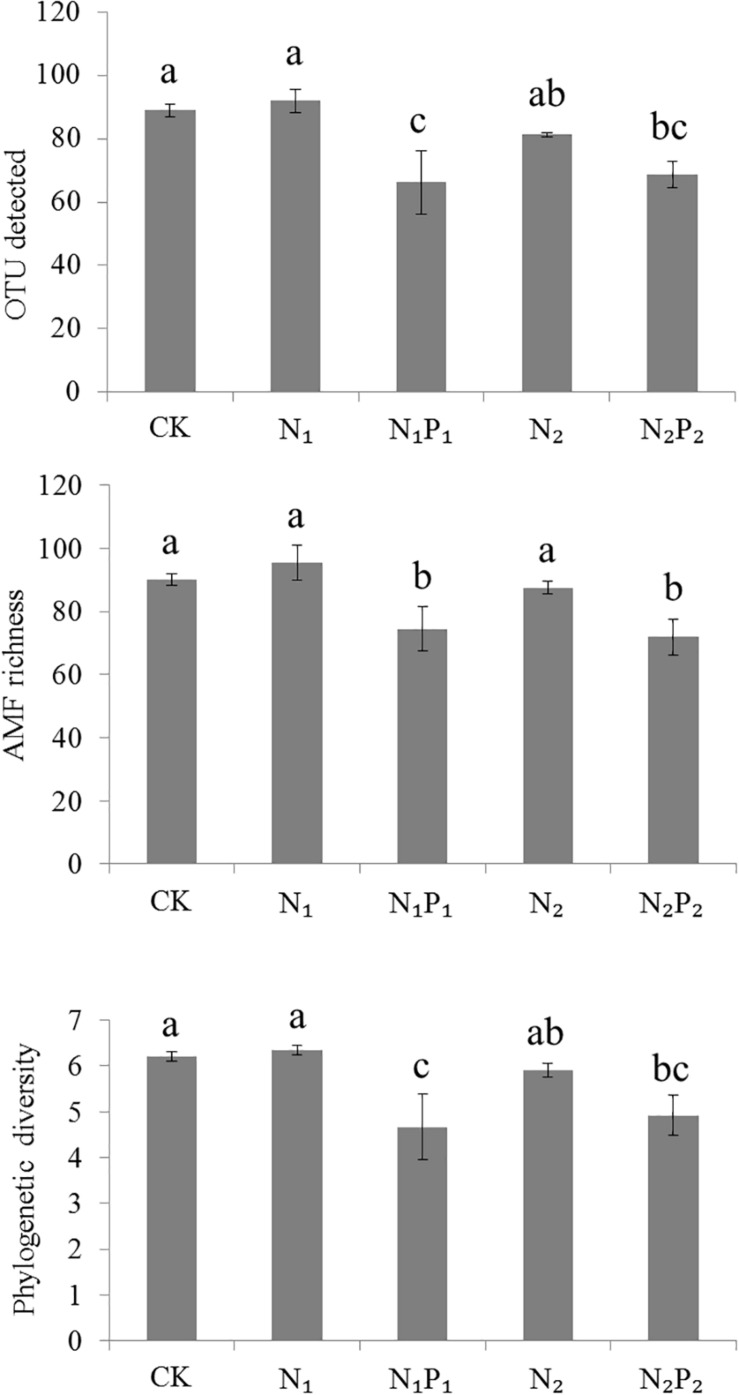
Alpha diversity of detected OTUs, AMF richness and phylogenetic diversity across soils from different fertilizer regimes. Vertical bars represent the standard deviations (*N* = 3) and the same letters above columns denote no significant difference (*P* < 0.05, Tukey’s test). Fertilizer regimes indicated as described in Table 1.

**FIGURE 2:**
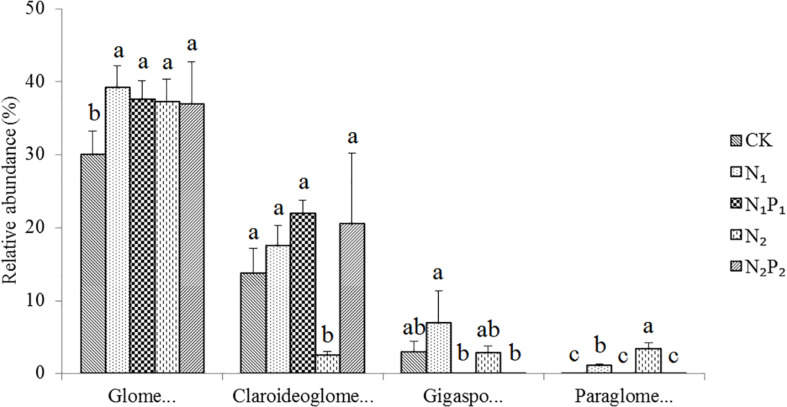
Relative abundance of arbuscular mycorrhizal fungal families (average abundance > 0.1%) for different long-term fertilizer treatments. Vertical bars represent the standard deviations (*N* = 3) and the same letters above columns denote no significant difference (*P* < 0.05, Tukey’s test). Fertilizer regimes indicated as described in Table 1.

The nested PCR successfully amplified DNA from all 15 samples. After quality control, a total of 409,049 high-quality sequences were obtained. They could be classified with a mean of 27,270 classifiable sequences per sample for use in the subsequent analysis (range 17,625–30,537). All the high-quality sequences were clustered into 220 OTUs; and 6551 sequences (clustered into 60 OTUs) represented non-AMF but this was only 1.6% of all sequences. The Good’s coverage values were all above 99.8% with a 97% similarity cutoff, indicating that the current depth of sequencing was sufficient to capture the AMF diversity. The numbers of OTUs in the five fertilizer treatments were in the range of 66–92 (Figure 1). In our study, 99.7% of all sequences matched Glomeromycota, and a total of 7 families were found in this study site. The four families (average abundance > 0.1%) Glomeraceae, Claroideoglomeraceae, Gigasporaceae, and Paraglomeraceae were represented in the sequencing dataset (Figure 2), the remaining three families Diversisporaceae, Archaeosporaceae, and Acaulosporaceae were low abundance in all rhizosphere soils. All soil samples were dominated by Glomeraceae, accounting for 30.0–39.1% of the sequences, followed by Claroideoglomeraceae (2.4–21.9%) and Gigasporaceae (0.03–2.8%). Family Paraglomeraceae was only occasionally detected at a low level. Moreover, 35–54% of sequences did not match any detailed taxonomic at family level.

**FIGURE 3:**
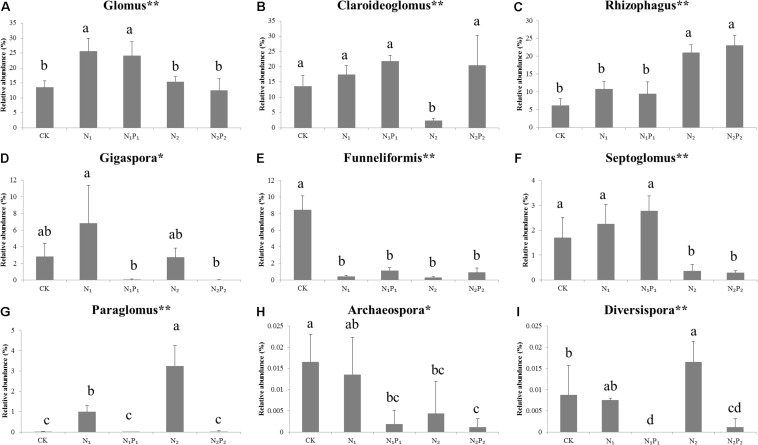
Relative abundance of arbuscular mycorrhizal fungal genera under different long-term fertilizer treatments. **(A)** Glomus; **(B)** Claroidegoglomus; **(C)** Rhizophagus; **(D)** Gigsspora; **(E)** Funneliformis; **(F)** Septoglomus; **(G)** Paraglomus; **(H)** Archaeospore; **(I)** Diversisppora. Vertical bars represent the standard deviations (*N* = 3) and the same letters above columns denote no significant difference (*P* < 0.05, Tukey’s test). **The difference is significant at the 0.01 level; * the difference is significant at the 0.05 level according to Tukey’s test. Fertilizer regimes indicated as described in Table 1.

**FIGURE 4:**
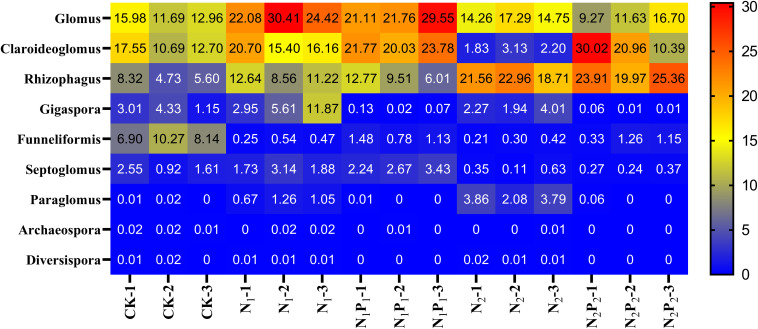
Heat map analysis the relative abundance of rhizosphere AMF genera after long term fertilization. Fertilizer regimes indicated as described in Table 1.

**FIGURE 5:**
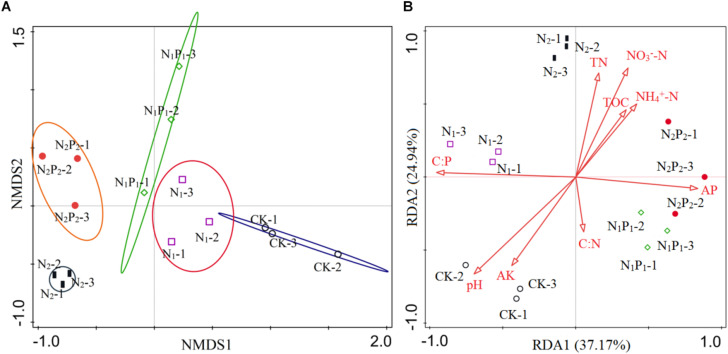
Non-metric multidimensional scaling (NMDS) ordination plot and redundancy analysis (RDA). NMDS shows changes in arbuscular mycorrhizal fungal (AMF) community compositions in long-term different fertilizer treatments **(A)**. RDA shows the relationship between AMF communities and soil parameters **(B)**. Soil factors indicated in red text include AP (available phosphorus), AK (available potassium), pH, NH_4_^+^-N (soil concentration of ammonium), NO_3_^–^-N (soil concentration of nitrate), TN (total nitrogen), TOC (total organic carbon), C:N (TOC:TN), C:P (TOC:AP).

Long-term fertilization significantly (*P* < 0.05) changed the abundance of families Glomeraceae, Claroideoglomeraceae, Gigasporaceae and Paraglomeraceae. The relative abundance of Glomeraceae in the N_1_, N_1_P_1_, N_2_ and N_2_P_2_ treatments was 9.2, 7.6, 7.2, and 6.9% higher, respectively, than that of CK. The relative abundance of Paraglomeraceae, which was only detected in N_1_ and N_2_ treatments, showed a positive relationship with N dosage (Figure 2). At genus level, the abundance of AMF distinctly responded to long-term N and P fertilization. The nine most abundant genera with significant differences are presented in Figures 3, 4. At genus level, *Glomus, Claroideoglomus*, and *Rhizophagus* were the dominant genera in wheat rhizosphere soil, occupying 18.26, 15.15, and 14.12% of the data set, respectively. The relative abundance of *Glomus* was increased by N_1_ and N_1_P_1_, but not significantly changed by N_2_ and N_2_P_2_, compared with CK. However, the relative abundance of *Rhizophagus* and *Septoglomus* was significantly increased and decreased by higher N addition (N_2_ and N_2_P_2_), respectively, but was not significantly changed by lower N addition (N_1_ and N_1_P_1_). Long-term fertilization decreased the relative abundance of *Funneliformis* and *Archaeospora.* For N_1_P_1_ and N_2_P_2_ treatments, the relative abundance of *Paraglomus* and *Diversispora* decreased to a greater extent than in the N_1_ and N_2_ treatments. AMF alpha diversity, including OTUs, AMF richness and phylogenetic diversity, was significant altered in the rhizosphere (Figure 1). The OTUs, phylogenetic diversity and AMF richness were lower in N_1_P_1_ and N_2_P_2_ treatments compared to CK, but there were no significant differences among CK, N_1_ and N_2_ treatments. AMF richness in N_1_P_1_ and N_2_P_2_ treatments was decreased by 17.3 and 20.2%, respectively, compared with CK. Beta-diversity analysis (NMDS) using Bray-Curtis distance suggested that the samples were well separated from each other. NMDS1 generally distributed the AMF communities along with soil pH: the AMF communities with acidic pH were generally in the left portion and those with higher pH to the right (Table 1 and Figure 5A). NMDS2 generally distributed the AMF communities along with soil AP: the AMF communities with high AP concentration were generally in the upper portion and those with low AP concentration in the lower portion.

### Relationships Between AMF Community and Environmental Variables

Nine variables were selected by RDA as predictors of species persistence (Figure 5B). All variables together explained 77.38% of the variation in AMF communities between samples. The first axis explained 37.17% of the data variation, and the second axis explained 24.94%. In Figure 5B, the lengths of the red arrows indicated the relative importance of each variable in explaining the rhizosphere AMF community composition, and soil C:P ratio, pH and AP were the three longest arrows. Indeed, based on this model, soil C:P ratio (*F* = 6.8, *P* = 0.002), pH (*F* = 5.5, *P* = 0.002), and AP (*F* = 3.6, *P* = 0.002) were the three most important contributors to the variation in AMF communities, and individually accounted for 34.2, 20.7, and 11.2% of variation, according to the Monte Carlo test, respectively. The first axis was associated with variables related to soil AP concentration and soil C:P ratio as reflected by the complete set of data. Soil C:P ratio was positive correlated with the rhizosphere AMF composition in CK and N_1_ treatments, whereas negative correlated with communities in N_1_P_1_ and N_2_P_2_. The oppose trend was found with soil AP. The soil pH changed by long-term fertilization was positive correlated with AMF composition in CK, whereas negative correlated with AMF composition in N_2_ and N_2_P_2_.

Pearson’s correlation analysis with AMF community composition in genera level and AMF diversity level confirms this relationship with soil C:P ratio, pH and AP. In diversity level, the OTUs, AMF richness and phylogenetic diversity indices were all significantly positively correlated with soil C:P ratio (*P* < 0.01), but negatively correlated with AP. However, soil pH was only significantly negatively correlated with OTUs and AMF richness (Supplementary Table S1).

In genera level, the soil C:P ratio was significantly positively correlated with relative abundance of *Septoglomus* and *Archaeospora*; soil pH was significantly positively correlated with the relative abundance of *Funneliformis*, *Septoglomus*, and *Archaeospora*, but negatively correlated with *Rhizophagus*; and AP concentration was positively correlated with *Rhizophagus*, but negatively correlated with *Septoglomus* and *Archaeospora.* In addition, soil C:N was also significantly correlated with *Glomus*, *Claroideoglomus*, *Rhizophagus*, and *Septoglomus*.

In this study, no genus was positively correlated with wheat yield; however, some genera were negatively correlated: *Gigaspora*, *Funneliformis*, *Archaeospora*, and *Diversispora* (Table 2).

**TABLE 2:** Pearson’s correlation coefficients for abundances among arbuscular mycorrhizal fungal genera, soil properties and wheat yield.

Genus		pH	AK	AP	NO_3_^–^-N	NH_4_^+^-N	TN	TOC	C:N	C:P	WY
*Glomus*	*R*	0.202	0.153	−0.291	−0.450	−0.466	−0.456	0.328	**0.616**	0.214	0.344
	*P*	0.471	0.587	0.293	0.092	0.080	0.088	0.233	0.015	0.443	0.210
*Claroideoglomus*	*R*	0.043	0.109	0.409	−0.425	−0.237	−0.472	0.383	**0.635**	−0.253	0.400
	*P*	0.878	0.700	0.130	0.114	0.396	0.076	0.159	0.011	0.362	0.140
*Rhizophagus*	*R*	**−0.902**	**−0.734**	**0.533**	**0.939**	**0.844**	**0.753**	0.306	**−0.516**	−0.453	0.228
	*P*	0.000	0.002	0.041	0.000	0.000	0.001	0.268	0.049	0.090	0.414
*Gigaspora*	*R*	0.346	−0.065	**−0.557**	−0.157	−0.116	0.049	−0.140	−0.125	**0.804**	−0.494
	*P*	0.207	0.818	0.031	0.575	0.682	0.863	0.618	0.657	0.000	0.061
*Funneliformis*	*R*	**0.753**	**0.626**	−0.270	−0.475	−0.401	−0.405	**−0.819**	−0.110	0.261	**−0.533**
	*P*	0.001	0.012	0.331	0.073	0.138	0.135	0.000	0.695	0.348	0.041
*Septoglomus*	*R*	**0.578**	**0.522**	−0.353	**−0.832**	**−0.761**	**−0.671**	0.065	**0.659**	0.172	−0.168
	*P*	0.024	0.046	0.197	0.000	0.001	0.006	0.817	0.008	0.540	0.550
*Paraglomus*	*R*	−0.313	−0.344	−0.396	0.502	0.278	**0.681**	0.154	−0.510	0.210	−0.305
	*P*	0.257	0.209	0.144	0.056	0.316	0.005	0.584	0.052	0.453	0.269
*Archaeospora*	*R*	**0.692**	0.273	**−0.525**	−0.442	−0.353	−0.141	−0.487	−0.146	**0.646**	**−0.588**
	*P*	0.325	0.004	0.066	0.325	0.044	0.099	0.197	0.616	0.009	0.021
*Diversispora*	*R*	0.090	0.037	**−0.547**	0.270	−0.013	0.354	−0.182	−0.425	0.461	**−0.631**
	*P*	0.751	0.896	0.035	0.331	0.962	0.196	0.516	0.114	0.084	0.012

*Bold values are significant at P < 0.05.*

### Integrated Responses of AMF Structure on Soil Properties and Plant

**FIGURE 6:**
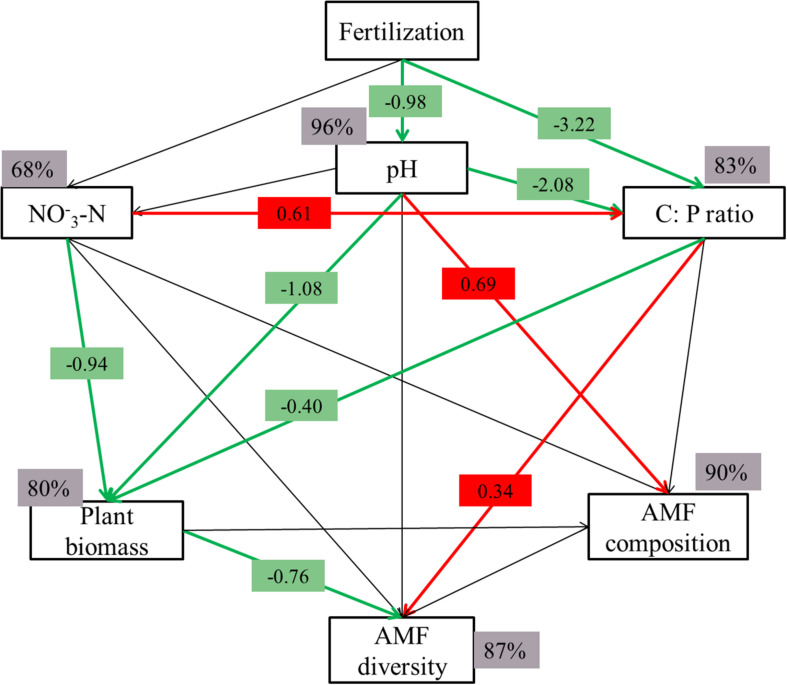
Structural equation modeling (SEM) of the relationships between soil properties (pH, C: P ratio and NO_3_-N), plant and AMF diversity and composition in wheat rhizosphere. The model resulted in a good fit to the data, with a model χ^2^/df = 0.888, *P* = 0.447, RESEA = 0.000, and GFI = 0.953. Red arrows indicate a positive correlation, while green indicates a negative relationship (*P* < 0.05). The numbers are the correlation coefficients. Percentages close to variables refer to the variance accounted for by the model (*R*^2^).

We also assessed the responses of AMF structure on soil properties and plant by employing a SEM model. The model proved a good fit to the data (χ^2^/df = 0.888; *P* = 0.447; RESEA = 0.000; GFI = 0.953), and accounted for 96% of the variation in pH, 68% and 83% in NO_3_^–^-N and C: P ratio, 80% in plant biomass, 87 and 90% in AMF composition and diversity, respectively (Figure 6). Fertilization significantly affected AMF diversity and composition due to decreased soil pH and C: P ratio and changed plant biomass (Figure 6). Decreased in soil C:P ratio and pH directly altered AMF diversity and AMF composition, respectively. In addition, alterations in plant biomass resulted in concomitant shifts in AMF diversity. Those results indicated that rhizosphere AMF structure was directly altered by soil property and indirectly shaped by changing plant biomass. The relationships between those variables were present in Supplementary Table S2, and some variables were not of great significance on their own, but they clearly improved the model fit when incorporated together.

## Discussion

In this study, we explored the AMF fungal communities in wheat rhizosphere soils. The analysis showed that long-term fertilization had great impacts on rhizosphere AMF species diversity and composition, and the rhizosphere AMF community structure was shaped both by fertilization and plant. The field resource in which the same fertilization regimes were conducted for 37 years enabled us to show that N and P addition produced a strong selective pressure against rhizosphere AMF over the long term in this study site.

### Long-Term Nutrient Addition Effects on Rhizosphere AMF Species Alpha-Diversity

In our study, N fertilizer did not significantly changed AMF diversity in the wheat rhizosphere, consistent with previous reports (Mueller and Bohannan, 2015; Dueñas et al., 2020). This may reflect the influence of interaction between fertilization and plant on rhizosphere AMF, as the relocation of C to AMF increased with N fertilization. For example, after a large N addition, relatively more plant derived ^13^C was allocation to AMF (Williams et al., 2017). In addition, Wang et al. (2019) found that long-term N fertilization increase the quantity of plant root exudates, which also modified the soil properties changes caused by N addition (Wang et al., 2018). Those results indicated that the stability of the AMF diversity after long-term N fertilization was due to both elevated fertilizer and host selection processes.

However, the N_1_P_1_ and N_2_P_2_ treatments had low AMF diversity in wheat rhizosphere soils than CK, consistent with a study of two grass species (Egerton-Warburton et al., 2007). In terms of OTUs detected and community composition, changes appeared due to the distinct species induced by those treatments, especially the taxa with lowest abundance (Figures 1–3). The loss of AMF species diversity has been reported to decrease both plant biodiversity and ecosystem productivity, and to increase ecosystem instability (Maček et al., 2011). The present study suggested AMF diversity decrease may result in a less stable agroecosystem and may lead to unsustainable crop production.

Egerton-Warburton et al. (2007) and Cheng et al. (2013) already found that changes to AMF diversity caused by N application may be linked to soil P availability; N fertilizer application cause reductions in P-rich soils. This indicated that N plus P addition may have a stronger influence on the rhizosphere AMF species diversity compared with only N addition (Figure 1). Although N fertilization increased plant allocate more C to AMF, N plus P fertilization decreased plant C allocation to AMF (Williams et al., 2017). Considering the most widely cited benefit of AMF is to enhance plant P acquisition (Hodge and Fitter, 2010), and more soil plant-available P reduce plant acquired P from AMF (Williams et al., 2017) and shift AMF functioning from mutualism toward parasitism (Jiang et al., 2018), some members of the rhizosphere AMF have greater carbon demands or have a less efficient use of carbon may be extinguished. This may explain why N plus P addition decreased rhizosphere AMF diversity compared with CK. In addition, we also found that there was no significance between N_2_ and N_2_P_2_ treatment on OTUs detected and Phylogenetic diversity (Figure 1). This may be attributing to high N application significantly decreased soil pH and not significantly increased plant biomass compared with low N application (Table 1), as rhizosphere AMF diversity was both shaped by soil pH and plant biomass (Figure 6). After a long time (such as 37 years) of sufficient P available and dramatic changes soil properties like soil pH (caused by long-term N fertilization), the AMF species that cannot acquire enough energy may be extinguished.

Overall, our results suggested that the responses of rhizosphere AMF to elevate fertilizer and plant changes with soil nutrients. When N fertilization increases N supply (P supply is the most limiting resource), the benefit of the symbiosis is enhanced. In this scenario, rhizosphere AMF diversity levels might be maintained. When N and P fertilization increases N and P supply, the benefit provided by this symbiosis is reduced (Dueñas et al., 2020). This may intensify competition among AMF taxa for plant derived C. In this case, a reduction in rhizosphere AMF diversity can be expected.

### Long-Term Nutrient Addition Changed Rhizosphere AMF Community Composition

The NMDS analysis demonstrated that AMF communities differed significantly among different fertilization regimes, suggesting that different N and P additions induced a shift in AMF community composition. These results agree with former findings that the abundance of soil nutrients plays a crucial role in shaping the rhizosphere AMF community (Qin et al., 2015; Dueñas et al., 2020). It was reported that nutrients accumulation caused markedly changes in soil chemical properties, species that are insensitive to the disturbances or efficient in acquiring plant C will increase in relative abundance (Koch et al., 2017; Wang et al., 2018). In addition, competition can also lead to the complete exclusion of particular species (Roger et al., 2013) or a decline in overall AMF abundance (Engelmoer et al., 2014). The addition of N plus P fertilization may select for taxa with better ability to hoard P in order to maximize C gains from the host (Whiteside et al., 2019).

In our study, we only found 7 AMF families, similar with the results of Zhu et al. (2020), which found 7 ∼ 8 families in different site of Chinese black soil region. However, Redecker et al. (2013) systematic reviewed of AMF taxa, and summarized 11 AMF families from literatures. The other four families were not detected including Sacculosporaceae, Pacisporaceae, Ambisporaceae, and Geosiphonaceae in our study site. This may be due to those four families were in low abundance, and cannot be detected by Illumina MiSeq platform. For example, Gu et al. (2020) found only less than 0.4% of all sequences belonged to Sacculosporaceae and Pacisporaceae in Chinese black soil. In addition, family Pacisporaceae showed a preferential distribution toward poor nutrient soils, rather than nutrient-rich black soil (Wang et al., 2018; Dudinszky et al., 2019). Cao et al. (2020) and Panneerselvam et al. (2020) found that Ambisporaceae and Geosiphonaceae were more abundant in higher temperature (mean annual temperature 19.1°C) and elevated CO_2_ concentration (550 μmol mol^–1^), respectively. The low temperature, atmospheric CO_2_ concentration and high nutrient level may explain why we did not detect those four families in our study site.

In the present study, we observed that long-term N and P fertilization increased the relative abundance of Glomeraceae compared with CK (Figure 2), with the relative Glomeraceae abundance was 39.1%, among them the relative genus *Glomus* abundance increased by 89.3% compared with CK (Figure 3A). Previous studies found that the family Glomeraceae could grant better protection from pathogens (Powell et al., 2009), as the majority biomass in the Glomeraceas is found in hyphae growing inside the root and reduce root infection of plant by two soil pathogens (Maherali and Klironomos, 2007). Those results indicated that N and P fertilization shifted the AMF communities toward disease-suppression against root pathogens. Our results also demonstrated that the relative abundance of Paraglomeraceae was higher in N_1_ and N_2_ treatments than other treatments (Figure 2), similar to observations in some previous studies (Hijri et al., 2006; Chagnon et al., 2013). The Paraglomeraceae with the majority of fungal biomass are located outside the plant were demonstrated to be more efficient in P assimilation (Powell et al., 2009). Those results indicated that long-term N (only) addition changed AMF communities by increasing nutrient assimilation taxa. The results also indicated that long-term N fertilization only may enhance plant P acquisition via AMF communities. In addition, Jiang et al. (2018) found that increased nutrients available would shift mycorrhizal functioning toward parasitism, and that the inefficient in acquiring soil derived C under elevated plant-available N and P levels in soil may be extinguished. However, the links between AMF traits and nutrient requirements or function inferred from a fraction of AMF isolates, and this may prevent us to unequivocally establish whether differential adaptations to nutrient supply are the basis for the patterns reported here.

### Factors Affecting Rhizosphere AMF Community Composition

The interactions among microbes, plants and soils play a critical role in ecosystem functioning (Zeng et al., 2016). However, earlier studies about the effects of fertilization on soil AMF communities did not include plant production data, often neglecting the interaction between elevated fertilizer and host selection processes on AMF communities. In our study, we found that N and P fertilization resulted in significant changes in rhizosphere AMF community composition. We further found that rhizosphere AMF composition was directly affected by decreasing soil pH caused by N and P fertilization, while AMF diversity was directly affected by soil C: P ratio and indirectly affected by plant biomass (Figure 6).

In our study, the composition of rhizosphere AMF communities was closely correlated with soil C:P (*P* < 0.05), as reported by Qin et al. (2015). This could be highly associated with AMF receiving their C supply from their host plants and compensating the plant through enhanced nutrient acquisition, particularly through supply of poorly mobile phosphate ions (Karasawa et al., 2012). In the case of greater nutrient sufficiency in plants in fertilizer treatments, symbiosis with AMF could be less important, reducing C allocation to AMF in the rhizosphere. This could explain why soil C:P was an important factor in shaping AMF communities, and indicated that rhizosphere AMF community was shaped by both soil nutrient and plant selection processes.

The shifts in the relative abundance of species taxonomic groups across different soil C:P values are similar to soil C:P responses observed in other studies. For example, the relative abundance of *Gigaspora* and *Archaeospora* had strong positive correlations with high soil C:P in this study (Table 2), consistent with results of Pagano and Scotti (2010). The relative abundances of *Gigaspora* and *Archaeospora* were higher in N_1_ and N_2_ than N_1_P_1_ and N_2_P_2_ treatments and this was significantly negatively correlated with AP concentration (*P* < 0.05). Previous studies demonstrated that P addition could shift species composition in favor of less efficient mutualists, which are competitively superior colonizers (Johnson, 1993). This indicated that the genera *Gigaspora* and *Archaeospora* were efficient mutualists with wheat in P-deficient soil in this site.

The soil pH was also an important factor affecting the relative abundance of species taxonomic groups, which was consistent with other reports (Jansa et al., 2014; Qin et al., 2015). In the present study, RDA also indicated that the AMF composition was affected by soil pH (Figure 6). The relative abundance of *Funneliformis*, *Septoglomus*, and *Archaeospora* were positively correlated with soil pH, but *Rhizophagus* was negatively correlated with it, as similarly reported by Jansa et al. (2014). With the increase in the amount of fertilizer, soil pH decreased from 6.81 to 5.45 (Table 1), and abundance of these AMF taxa changed gradually. The results indicated that these AMF groups were sensitive to soil pH. *Rhizophagus* was relatively more abundant in N_2_ and N_2_P_2_ than other treatments (Figures 3, 4), and positively correlated with most available nutrients (Table 2). This is may be because *Rhizophagus* can flourish in soils with large amounts of nutrients, and exert a P-uptake function with a large requirement of energy source (Thonar et al., 2011). The available nutrients were higher in N_2_ and N_2_P_2_ than other fertilizer treatments and CK (Table 1). The relative abundances of *Glomus* and *Claroideoglomus* were significantly positively correlated with soil C:N values, consistent with other reports that soil C:N was also an important factor affecting the soil AMF community (Shi et al., 2014; Treseder et al., 2018; Dueñas et al., 2020).

## Conclusion

We demonstrated that rhizosphere AMF species diversity was decreased by N plus P fertilization, but not significantly affected by fertilization with N only. Composition of the rhizosphere AMF community was significantly influenced by N and P fertilizers, which appeared to be mediated greatly by soil-plant-microbe interactions. Specifically, the rhizosphere AMF diversity was directly affected by soil C: P ratio and indirectly affected by plant biomass, while rhizosphere AMF community was directly shaped by soil acidification. Our results suggest that rhizosphere AMF community structure was shaped by elevated fertilizer and host selection processes. The 37-year inorganic fertilization regimes changed rhizosphere AMF communities with a potential negative impact on AMF transport of nutrients to plants and on the beneficial effect of AMF genera to plant resistance against pathogens, because long-term inorganic fertilization promotes microbes with known pathogenic traits (Zhou et al., 2016). More studies need to be conducted to elucidate the mechanisms that AMF taxa and communities use to cope with the pathogens and nutrients transportation caused by global environmental changes, such as N- or P-deposition. Most importantly, studies about how soil-plant-microbe interactions influence on ecosystem functions and progresses remains to be elucidated.

## Data Availability Statement

The datasets generated for this study can be found in the NCBI Sequence Read Archive, SRX3008208.

## Author Contributions

QW, SW, and JL conceptualized and designed the experiments. MM, XJ, DG, FC, YK, and CC analyzed the data. DW provided the material for this experiment. All authors contributed to the article and approved the submitted version.

## Conflict of Interest

The authors declare that the research was conducted in the absence of any commercial or financial relationships that could be construed as a potential conflict of interest.

## References

[B1] Camenzind T. Hempel S. Homeier J. Horn S. Velescu A. Wilcke W. (2014). Nitrogen and phosphorus additions impact arbuscular mycorrhizal abundance and molecular diversity in a tropical montane forest. *Glob. Change Biol.* 20 3646–3659. 10.1111/gcb.12618 24764217

[B2] Cao J. Lin T. C. Yang Z. Zheng Y. Xie L. Xiong D. (2020). Warming exerts a stronger effect than nitrogen addition on the soil arbuscular mycorrhizal fungal community in a young subtropical cunninghamia lanceolata plantation. *Geoderma* 367:114273 10.1016/j.geoderma.2020.114273

[B3] Chagnon P. L. Bradley R. L. Maherali H. Klironomos J. N. (2013). A trait-based framework to understand life history of mycorrhizal fungi. *Trends Plant Sci.* 18 484–491. 10.1016/j.tplants.2013.05.001 23756036

[B4] Cheng Y. Ishimoto K. Kuriyama Y. Osaki M. Ezawa T. (2013). Ninety- year-, but not single, application of phosphorus fertilizer has a major impact on arbuscular mycorrhizal fungal communities. *Plant Soil* 365 397–407. 10.1007/s11104-012-1398-x

[B5] Dudinszky N. Cabello M. N. Grimoldi A. A. Schalamuk S. Golluscio R. A. (2019). Role of grazing intensity on shaping arbuscular mycorrhizal fungi communities in patagonian semiarid steppes. *Rangeland Ecol. Manag.* 72 692–699. 10.1016/j.rama.2019.02.007

[B6] Dueñas J. F. Camenzind T. Roy J. Hempel S. Homeier J. Suárez J. P. (2020). Moderate phosphorus additions consistently affect community composition of arbuscular mycorrhizal fungi in tropical montane forests in southern Ecuador. *New Phytol.* 227 1505–1518. 10.1111/nph.16641 32368801

[B7] Edgar R. C. (2013). UPARSE: highly accurate OTU sequences from microbial amplicon reads. *Nat. Methods* 10 996–998. 10.1038/nmeth.2604 23955772

[B8] Egerton-Warburton L. M. Allen E. B. (2000). Shifts in arbuscular mycorrhizal communities along an anthropogenic nitrogen deposition gradient. *Ecol. Appl.* 10 484–496. 10.1890/1051-0761(2000)010[0484:siamca]2.0.co;2

[B9] Egerton-Warburton L. M. Johnson N. C. Allen E. B. (2007). Mycorrhizal community dynamics following nitrogen fertilization: a cross-site test in five grasslands. *Ecol.Monogr.* 77 527–544. 10.1890/06-1772.1

[B10] Engelmoer D. J. Behm J. E. Toby Kiers E. (2014). Intense competition between arbuscular mycorrhizal mutualists in an in vitro root microbiome negatively affects total fungal abundance. *Mol. Ecol.* 23 1584–1593. 10.1111/mec.12451 24050702

[B11] Ericsson T. (1995). Growth and shoot: root ratio of seedlings in relation to nutrient availability. *Plant Soil* 168 205–214. 10.1007/978-94-011-0455-5_23

[B12] Gollotte A. van Tuinen D. Atkinson D. (2004). Diversity of arbuscular mycorrhizal fungi colonising roots of the grass species *Agrostis capillaris* and *Lolium perenne* in a field experiment. *Mycorrhiza* 14 111–117. 10.1007/s00572-003-0244-7 12768382

[B13] Gu S. Wu S. Guan Y. Zhai C. Zhang Z. Bello A. (2020). Arbuscular mycorrhizal fungal community was affected by tillage practices rather than residue management in black soil of northeast China. *Soil Till. Res.* 198:104552 10.1016/j.still.2019.104552

[B14] Helmke P. A. Sparks D. (1996). “Lithium, sodium, potassium, rubidium, and cesium,” in *Methods of Soil Analysis Part 3—Chemical Methods and Process*, ed. Sparks D. L. (Madison, WI: SSSA), 551–574.

[B15] Hijri I. Sykorova Z. Oehl F. Ineichen K. Mader P. Wiemken A. (2006). Communities of arbuscular mycorrhizal fungi in arable soils are not necessarily low in diversity. *Mol. Ecol.* 15 2277–2289. 10.1111/j.1365-294x.2006.02921.x 16780440

[B16] Hodge A. Fitter A. H. (2010). Substantial nitrogen acquisition by arbuscular mycorrhizal fungi from organic material has implications for N cycling. *Proc. Natl. Acad. Sci. U.S.A.* 107 13754–13759. 10.1073/pnas.1005874107 20631302 PMC2922220

[B17] Jansa J. Erb A. Oberholzer H. R. Smilauer P. Egli S. (2014). Soil and geography are more important determinants of indigenous arbuscular mycorrhizal communities than management practices in Swiss agricultural soils. *Mol. Ecol.* 23 2118–2135. 10.1111/mec.12706 24611988

[B18] Jiang S. Liu Y. Luo J. Qin M. Johnson N. Öpik M. (2018). Dynamics of arbuscular mycorrhizal fungal community structure and functioning along a nitrogen enrichment gradient in an alpine meadow ecosystem. *New Phytol.* 220 1222–1235. 10.1111/nph.15112 29600518

[B19] Johnson N. C. (1993). Can fertilization of soil select less mutualistic mycorrhizae? *Ecol. Appl.* 3 749–757. 10.2307/1942106 27759303

[B20] Johnson N. C. (2010). Resource stoichiometry elucidates the structure and function of arbuscular mycorrhizas across scales. *New Phytol.* 185 631–647. 10.1111/j.1469-8137.2009.03110.x 19968797

[B21] Karasawa T. Hodge A. Fitter A. (2012). Growth, respiration and nutrient acquisition by the arbuscular mycorrhizal fungus *Glomus mosseae* and its host plant *Plantago lanceolata* in cooled soil. *Plant Cell Environ.* 35 819–828. 10.1111/j.1365-3040.2011.02455.x 22070553

[B22] Koch A. M. Antunes P. M. Maherali H. Hart M. M. Klironomos J. N. (2017). Evolutionary asymmetry in the arbuscular mycorrhizal symbiosis: conservatism in fungal morphology does not predict host plant growth. *New Phytol.* 214 1330–1337. 10.1111/nph.14465 28186629

[B23] Lin X. Feng Y. Zhang H. Chen R. Wang J. Zhang J. (2012). Long-term balanced fertilization decreases arbuscular mycorrhizal fungal diversity in an arable soil in north china revealed by 454 pyrosequencing. *Environ. Sci. Technol.* 46 5764–5771. 10.1021/es3001695 22582875

[B24] Maherali H. Klironomos J. N. (2007). Influence of phylogeny on fungal community assembly and ecosystem functioning. *Science* 316, 1746–1748. 10.1126/science.1143082 17588930

[B25] Maček I. Dumbrell A. J. Nelson M. Fitter A. H. Vodnik D. Helgason T. (2011). Local adaptation to soil hypoxia determines the structure of an arbuscular mycorrhizal fungal community in roots from natural CO2 springs. *Appl. Environ. Microbiol.* 77 4770–4777. 10.1128/aem.00139-11 21622777 PMC3147400

[B26] Mueller R. C. Bohannan B. J. (2015). Shifts in the phylogenetic structure of arbuscular mycorrhizal fungi in response to experimental nitrogen and carbon dioxide additions. *Oecologia* 179 175–185. 10.1007/s00442-015-3337-z 25990297

[B27] Mummey D. L. (2006). Mycorrhizas and soil structure. *New Phytol.* 171 41–53. 10.1111/j.1469-8137.2006.01750.x 16771981

[B28] Ning Q. Chen L. Jia Z. Zhang C. Ma D. Li F. (2020). Multiple long-term observations reveal a strategy for soil pH-dependent fertilization and fungal communities in support of agricultural production. *Agric. Ecosyst. Environ.* 293 106837–106846. 10.1016/j.agee.2020.106837

[B29] Olsen S. R. (1954). *Estimation of Available Phosphorus in Soils by Extraction With Sodium Bicarbonate.* Washington, DC: United States Department Of Agriculture.

[B30] Pagano M. C. Scotti M. R. (2010). Effect of phosphorus fertilization on arbuscular mycorrhizal colonization of *Zeyheria tuberculosa* a native species in Brazil’s forest. *Middle East J. Sci. Res.* 6 604–611.

[B31] Panneerselvam P. Kumar U. Senapati A. Parameswaran C. Anandan A. Kumar A. (2020). Influence of elevated CO2 on arbuscular mycorrhizal fungal community elucidated using Illumina MiSeq platform in sub-humid tropical paddy soil. *Appl. Soil Ecol.* 145:103344 10.1016/j.apsoil.2019.08.006

[B32] Powell J. R. Parrent J. L. Hart M. M. Klironomos J. N. Rillig M. C. Maherali H. (2009). Phylogenetic trait conservatism and the evolution of functional trade-offs in arbuscular mycorrhizal fungi. *Proc. Biol. Sci.* 276 4237–4245. 10.1098/rspb.2009.1015 19740877 PMC2821337

[B33] Qin H. Lu K. Strong P. Xu Q. Wu Q. Xu Z. (2015). Long-term fertilizer application effects on the soil, root arbuscular mycorrhizal fungi and community composition in rotation agriculture. *Appl. Soil Ecol.* 89 35–43. 10.1016/j.apsoil.2015.01.008

[B34] Redecker D. Schüßler A. Stockinger H. Stürmer S. L. Morton J. B. Walker C. (2013). An evidence-based consensus for the classification of arbuscular mycorrhizal fungi (Glomeromycota). *Mycorrhiza* 23 515–531. 10.1007/s00572-013-0486-y 23558516

[B35] Roger A. Colard A. Angelard C. Sanders I. R. (2013). Relatedness among arbuscular mycorrhizal fungi drives plant growth and intraspecific fungal coexistence. *ISME J.* 7 2137–2146. 10.1038/ismej.2013.112 23823490 PMC3806264

[B36] Schloss P. D. Westcott S. L. Ryabin T. Hall J. R. Hartmann M. Hollister E. B. (2009). Introducing mothur: open-source, platform-independent, community-supported software for describing and comparing microbial communities. *Appl. Environ. Microbiol.* 75 7537–7541. 10.1128/aem.01541-09 19801464 PMC2786419

[B37] Schmidt J. E. Kent A. D. Brisson V. L. Gaudin A. C. (2019). Agricultural management and plant selection interactively affect rhizosphere microbial community structure and nitrogen cycling. *Microbiome* 7 1–18. 31699148 10.1186/s40168-019-0756-9 PMC6839119

[B38] Shi G. Liu Y. Johnson N. C. Olsson P. A. Lin M. Cheng G. (2014). Interactive influence of light intensity and soil fertility on root-associated arbuscular mycorrhizal fungi. *Plant Soil* 378 173–188. 10.1007/s11104-014-2022-z

[B39] Strickland T. Sollins P. (1987). Improved method for separating light-and heavy-fraction organic material from soil. *Soil Sci. Soc. Am. J.* 51 1390–1393. 10.2136/sssaj1987.03615995005100050056x

[B40] Thonar C. Schnepf A. Frossard E. Roose T. Jansa J. (2011). Traits related to differences in function among three arbuscular mycorrhizal fungi. *Plant Soil* 339 231–245. 10.1007/s11104-010-0571-3

[B41] Treseder K. K. Allen E. B. Egerton-Warburton L. M. Hart M. M. Klironomos J. N. Maherali H. (2018). Arbuscular mycorrhizal fungi as mediators of ecosystem responses to nitrogen deposition: a trait-based predictive framework. *J. Ecol.* 106 480–489. 10.1111/1365-2745.12919

[B42] Van Tuinen D. Jacquot E. Zhao B. Gollotte A. Gianinazzi-Pearson V. (1998). Characterization of root colonization profiles by a microcosm community of arbuscular mycorrhizal fungi using 25S rDNA-targeted nested PCR. *Mol. Ecol.* 7 879–887. 10.1046/j.1365-294x.1998.00410.x 9691489

[B43] Verbruggen E. Kiers E. T. Bakelaar P. N. Röling W. F. van der Heijden M. G. (2012). Provision of contrasting ecosystem services by soil communities from different agricultural fields. *Plant Soil* 350 43–55. 10.1007/s11104-011-0828-5

[B44] Wang Q. Jiang X. Guan D. Wei D. Zhao B. Ma M. (2018). Long-term fertilization changes bacterial diversity and bacterial communities in the maize rhizosphere of Chinese Mollisols. *Appl. Soil Ecol.* 125 88–96. 10.1016/j.apsoil.2017.12.007

[B45] Wang Q. Ma M. Jiang X. Zhou B. Guan D. Cao F. (2019). Long-term N fertilization altered 13C-labeled fungal community composition but not diversity in wheat rhizosphere of Chinese black soil. *Soil Biol. Biochem.* 135 117–126. 10.1016/j.soilbio.2019.04.009

[B46] Wei D. Yang Q. Zhang J. Z. Wang S. Chen X. Zhang X. L. (2008). Bacterial community structure and diversity in a black soil as affected by long-term fertilization. *Pedosphere* 18 582–592. 10.1016/s1002-0160(08)60052-1

[B47] Whiteside M. D. Werner G. D. Caldas V. E. van’t Padje A. Dupin S. E. Elbers B. (2019). Mycorrhizal fungi respond to resource inequality by moving phosphorus from rich to poor patches across networks. *Curr. Biol.* 29 2043–2050. 10.1016/j.cub.2019.04.061 31178314 PMC6584331

[B48] Williams A. Manoharan L. Rosenstock N. P. Olsson P. A. Hedlund K. (2017). Long-term agricultural fertilization alters arbuscular mycorrhizal fungal community composition and barley (*Hordeum vulgare*) mycorrhizal carbon and phosphorus exchange. *New Phytol.* 213 874–885. 10.1111/nph.14196 27643809

[B49] Xing B. Liu X. Liu J. Han X. (2005). Physical and chemical characteristics of a typical Mollisol in China. *Comm. Soil Sci. Plant Anal.* 35 1829–1838. 10.1081/lcss-200026802

[B50] Yin C. Fan F. Song A. Cui P. Li T. Liang Y. (2015). Denitrification potential under different fertilization regimes is closely coupled with changes in the denitrifying community in a black soil. *Appl. Microbiol. Biotechnol.* 99 5719–5729. 10.1007/s00253-015-6461-0 25715781

[B51] Zeng J. Liu X. Song L. Lin X. Zhang H. Shen C. (2016). Nitrogen fertilization directly affects soil bacterial diversity and indirectly affects bacterial community composition. *Soil Biol. Biochem.* 92 41–49. 10.1016/j.soilbio.2015.09.018

[B52] Zhou J. Jiang X. Zhou B. Zhao B. Ma M. Guan D. (2016). Thirty four years of nitrogen fertilization decreases fungal diversity and alters fungal community composition in black soil in northeast China. *Soil Biol. Biochem.* 95 135–143. 10.1016/j.soilbio.2015.12.012

[B53] Zhu X. Yang W. Song F. Li X. (2020). Diversity and composition of arbuscular mycorrhizal fungal communities in the cropland black soils of China. *Globle Ecol. Conserv.* 22:e00964 10.1016/j.gecco.2020.e00964

