# Supplementary material for: Influence of 37 Years of Nitrogen and Phosphorus Fertilization on Composition of Rhizosphere Arbuscular Mycorrhizal Fungi Communities in Black Soil of Northeast China
Source: Front Microbiol. 2020 Sep 8;11:539669. doi: 10.3389/fmicb.2020.539669 (PMC7506078; doi:10.3389/fmicb.2020.539669)
Supplement: Supplementary file 1 [file Data_Sheet_1.docx]

**
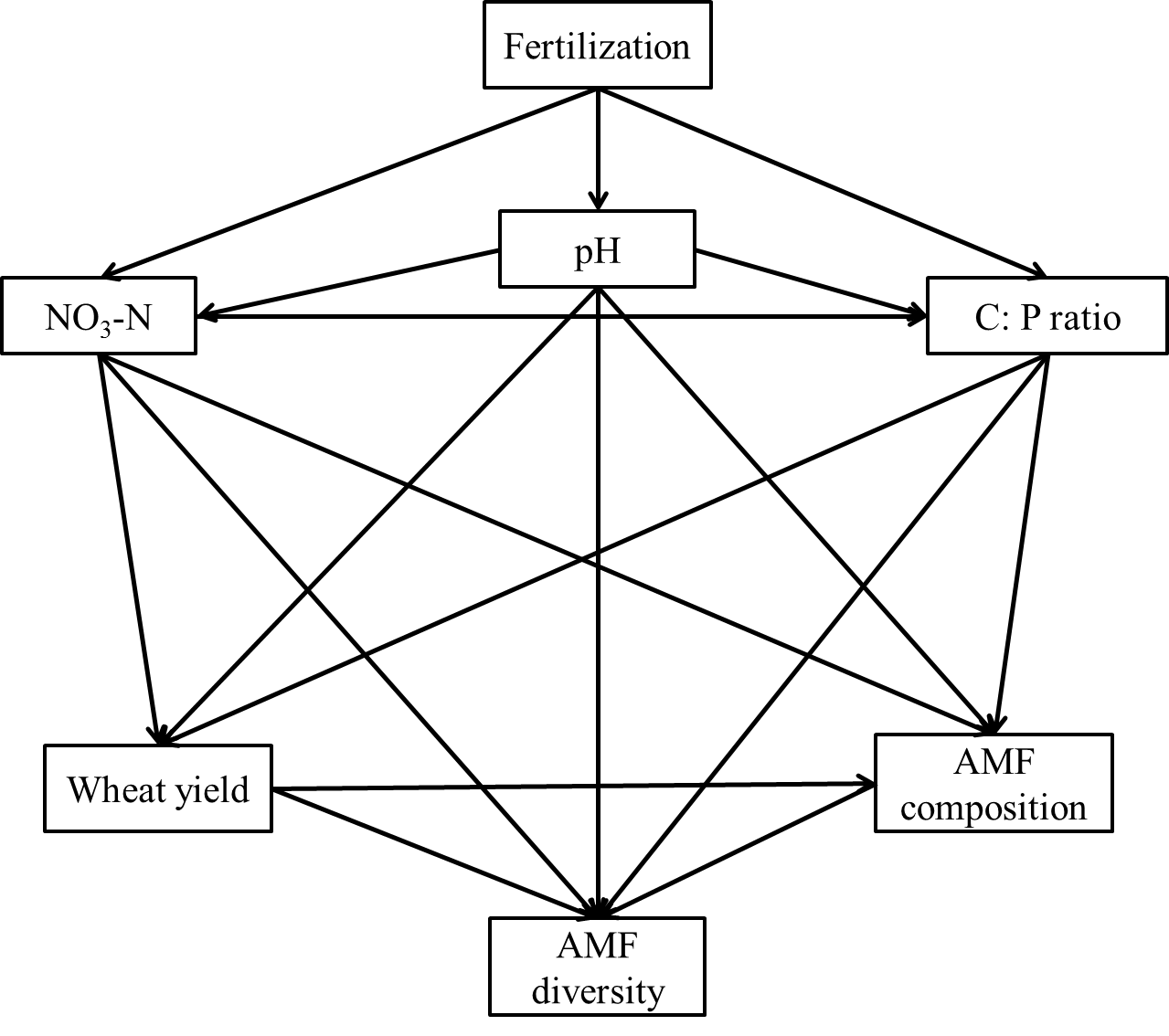
**

**Fig. S1.** The plausible interaction pathways in the structural equation model.

**Table S1**

Pearson’s correlation coefficients of wheat yield and AMF alpha diversity to soil properties.

| **Soil properties** |  | **AMF richness** | **OTUs^1^ detected** | **Phylogenetic diversity** | **WY** |
| --- | --- | --- | --- | --- | --- |
| **pH** | *R*  *P* | **0.575**  0.025 | **0.597**  0.019 | 0.500  0.058 | **−0.551**  0.033 |
| **AK** | *R*  *P* | 0.408  0.131 | 0.411  0.128 | 0.349  0.203 | −0.415  0.124 |
| **AP** | *R*  *P* | **−0.772**  0.001 | **−0.714**  0.003 | **−0.724**  0.002 | **0.663**  0.007 |
| **NO_3_^−^-N** | *R*  *P* | −0.258  0.354 | −0.220  0.430 | −0.113  0.689 | 0.092  0.745 |
| **NH_4_^+^-N** | *R*  *P* | −0.370  0.175 | −0.304  0.270 | −0.248  0.373 | 0.179  0.523 |
| **TN** | *R*  *P* | −0.062  0.827 | −0.072  0.798 | 0.047  0.869 | −0.089  0.754 |
| **TOC** | *R*  *P* | −0.188  0.501 | −0.334  0.224 | −0.257  0.355 | 0.479  0.071 |
| **C:N** | *R*  *P* | −0.051  0.858 | −0.133  0.637 | −0.200  0.474 | 0.362  0.185 |
| **C:P** | *R*  *P* | **0.835**  0.000 | **0.838**  0.000 | **0.808**  0.000 | **−0.740**  0.002 |

^1^OTUs: operational taxonomic units (97% similarity).

Fertilizer regimes: CK (without fertilizer), N_1_ (150 kg N ha^−1^), N_1_P_1_ (150 kg N ha^−1^ plus 75 kg P ha^−1^), N_2_ (300 kg N ha^−1^) and N_2_P_2_ (300 kg N ha^−1^plus 150 kg P ha^−1^).

Soil properties indicated include AP (available P), AK (available K), TOC (total organic carbon), TN (total N), C:N (TOC:TN), C:P (TOC:AP) and WY (wheat yield).

Bold values are significant at *P* < 0.05

**Table S2.**

Results of structural equation modeling of responses of AMF structure on soil properties and plant shown in Fig. 5.

|  |  |  | Estimate | Std Error | z value | P |
| --- | --- | --- | --- | --- | --- | --- |
| pH | <--- | Fertilization | -0.330 | 0.017 | -19.088 | **<0.001** |
| NO_3_^−^-N | <--- | Fertilization | 0.423 | 1.419 | 0.298 | 0.766 |
| NO_3_^−^-N | <--- | pH | -3.230 | 4.217 | -0.766 | 0.444 |
| C: P ratio | <--- | Fertilization | -2711.631 | 476.537 | -5.690 | **<0.001** |
| C: P ratio | <--- | pH | -5141.340 | 1441.320 | -3.567 | **<0.001** |
| C: P ratio | <--- | NO_3_^−^-N | 281.223 | 89.496 | 3.142 | **0.002** |
| Wheat yield | <--- | C: P ratio | -0.257 | 0.103 | -2.490 | **0.013** |
| Wheat yield | <--- | NO_3_^−^-N | -280.204 | 67.396 | -4.158 | **<0.001** |
| Wheat yield | <--- | pH | -1752.170 | 431.089 | -4.065 | **<0.001** |
| AMF composition | <--- | C: P ratio | 0.000 | 0.000 | -.282 | 0.778 |
| AMF composition | <--- | pH | 1.119 | 0.460 | 2.434 | **0.015** |
| AMF composition | <--- | NO_3_^−^-N | -0.086 | 0.073 | -1.184 | 0.237 |
| AMF composition | <--- | Wheat yield | 0.000 | 0.000 | -0.386 | 0.699 |
| AMF diversity | <--- | pH | -0.031 | 0.613 | -0.050 | 0.960 |
| AMF diversity | <--- | Wheat yield | -0.001 | 0.000 | -3.427 | **<0.001** |
| AMF diversity | <--- | composition | -0.228 | 0.299 | -0.763 | 0.445 |
| AMF diversity | <--- | NO_3_^−^-N | -0.043 | 0.085 | -0.500 | 0.617 |
| AMF diversity | <--- | C: P ratio | 0.000 | 0.000 | 2.129 | **0.033** |

Boldface indicates significant level for the regression weight.
